# Functional Glycosylation of Dystroglycan Is Crucial for Thymocyte Development in the Mouse

**DOI:** 10.1371/journal.pone.0009915

**Published:** 2010-03-29

**Authors:** Li-Ying Liou, Kevin B. Walsh, Arineh R. Vartanian, Daniel Beltran-Valero de Bernabe, Megan Welch, Kevin P. Campbell, Michael B. A. Oldstone, Stefan Kunz

**Affiliations:** 1 Viral-Immunobiology Laboratory, Department of Immunology and Microbial Science, The Scripps Research Institute, La Jolla, California, United States of America; 2 Howard Hughes Medical Institute, Department of Molecular Physiology and Biophysics, Departments of Neurology and Internal Medicine, University of Iowa Roy J. and Lucille A. Carver College of Medicine, Iowa City, Iowa, United States of America; 3 Department of Infectology, Scripps Florida, Jupiter, Florida, United States of America; 4 Institute of Microbiology, University Hospital Center and University of Lausanne, Lausanne, Switzerland; National Institute on Aging, United States of America

## Abstract

**Background:**

Alpha-dystroglycan (α-DG) is a cell surface receptor providing a molecular link between the extracellular matrix (ECM) and the actin-based cytoskeleton. During its biosynthesis, α-DG undergoes specific and unusual O-glycosylation crucial for its function as a high-affinity cellular receptor for ECM proteins.

**Methodology/Principal Findings:**

We report that expression of functionally glycosylated α-DG during thymic development is tightly regulated in developing T cells and largely confined to CD4^−^CD8^−^ double negative (DN) thymocytes. Ablation of DG in T cells had no effect on proliferation, migration or effector function but did reduce the size of the thymus due to a significant loss in absolute numbers of thymocytes. While numbers of DN thymocytes appeared normal, a marked reduction in CD4^+^CD8^+^ double positive (DP) thymocytes occurred. In the periphery mature naïve T cells deficient in DG showed both normal proliferation in response to allogeneic cells and normal migration, effector and memory T cell function when tested in acute infection of mice with either lymphocytic choriomeningitis virus (LCMV) or influenza virus.

**Conclusions/Significance:**

Our study demonstrates that DG function is modulated by glycosylation during T cell development *in vivo* and that DG is essential for normal development and differentiation of T cells.

## Introduction

Alpha-dystroglycan (α-DG) is an important cell surface receptor with high affinity for the extracellular matrix (ECM) proteins laminin, agrin, perlecan, and neurexin [1] and serves as a cellular receptor for several arenavirises, including lymphocytic choriomeningitis virus (LCMV) and the pathogenic Lassa virus (Cao et al., 1998). DG is encoded as a single polypeptide chain that is cleaved into the extracellular α-DG and membrane anchored β-DG which provides an essential molecular link between ECM and the actin-based cytoskeleton [2], [3]. A remarkable feature of α-DG is its complex pattern of post-translational modifications, including unusual sugar modifications, which are crucial for binding to ECM proteins, such as laminins, perlecan, and neurexins [1]. While the core protein of DG is ubiquitously expressed, functional glycosylation of α-DG is regulated both in the developing and adult organism. The genes of the known and putative glycosyltransferases POMT1/2, POMGnT1, LARGE1, LARGE2, fukutin and FKRP, which are involved in α-DG modification are affected in human congenital neuromuscular diseases, so-called “secondary dystroglycanopathies”, that manifest in a range of muscular and brain abnormalities caused by a loss of function of DG as an ECM receptor [1], [4], [5]. While the function of DG in muscle, brain, and other organs becomes increasingly clear, the role of this important ECM receptor in the immune system is largely unknown. Significant levels of DG core protein without detectable functional glycosylation are found in non-adherent cell types like resting blood lymphocytes [6]. The core protein of α-DG was found up-regulated after T cell activation and clustered at the immunological synapse (IS) formed between T cells and antigen-presenting cells *in vitro*, suggesting a function for α-DG in T cells [7] although its role *in vivo* was unclear. *In vivo*, the extent of functional glycosylation, which is crucial for α-DG's interaction with all known ligands has been examined in dendritic cells [8], [9], but not yet in T cells. In our present study we sought to close this gap by addressing the expression of functionally glycosylated α-DG during T cell development and investigating the function of DG in developing and mature T cells *in vivo*.

Unlike the majority of other hematopoietic cell lineages that mature in the bone marrow, T cell progenitors leave the bone marrow and migrate through the blood stream to another primary lymphatic organ, the thymus, for further development [10], [11], [12]. Once in the thymus, T cell progenitors proceed through a series of stages of cell proliferation and differentiation that are tightly controlled at multiple checkpoints associated with differential gene expression and regulation [13], [14]. Early CD4^−^CD8^−^ double negative (DN) thymocytes undergo a complex pattern of migration and differentiation characterized by sequential changes in expression of the cell surface markers CD25 and CD44 through the stages DN1 (CD25^−^CD44^+^), DN2 (CD25^+^CD44^+^), DN3 (CD25^+^CD44^−^), and DN4 (CD25^−^CD44^−^). At the DN3 stage, successful rearrangement of T cell receptor (TCR) β subunit genes results in formation of a pre-TCR complex that induces ligand-independent activation signals for thymocyte survival, proliferation, and differentiation into CD4^+^CD8^+^ double positive (DP) thymocytes, a process known as β-selection [10], [11], [12], [15]).

The migration and differentiation of T cell progenitors in the thymus is highly regulated and critically depends on the interaction of thymocytes with ECM and other cell types including thymic stromal cells and/or dendritic cells. ECM associated with thymic epithelial cells (TEC) of the subcapsular region where DN3 cells undergo β-selection is rich in laminin isoforms containing the laminin α2 chain (laminin-2/4) or the α5 chain (laminin-10/11) [16], [17] that undergo high affinity interactions with α-DG. The presence of these α-DG ligands in thymic ECM suggests that cell-matrix interactions mediated by DG may play a role in T cell survival and/or differentiation.

In the present study, we examined the expression of functionally glycosylated α-DG during T cell development and investigated the function of DG in developing and mature T cells *in vivo*. To address the role of α-DG in T cells *in vivo*, we conditionally deleted the *DG* core protein in developing thymocytes using *Cre-loxP* methodology. Ablation of DG in T cells resulted in reduced size of the thymus due to a significant reduction in absolute number of thymocytes. While numbers of DN thymocytes appeared normal, a marked reduction in DP thymocytes was observed, accompanied with a significant increase of apoptosis in the thymus. Remarkably, mature naïve T cells deficient in DG showed normal migration pattern and effector function when challenged with either LCMV, or influenza virus as well as a normal proliferative response to allogeneic stimulation.

## Results

### Functionally Glycosylated α-DG is Differentially Expressed During T Cell Development

**Figure 1:**
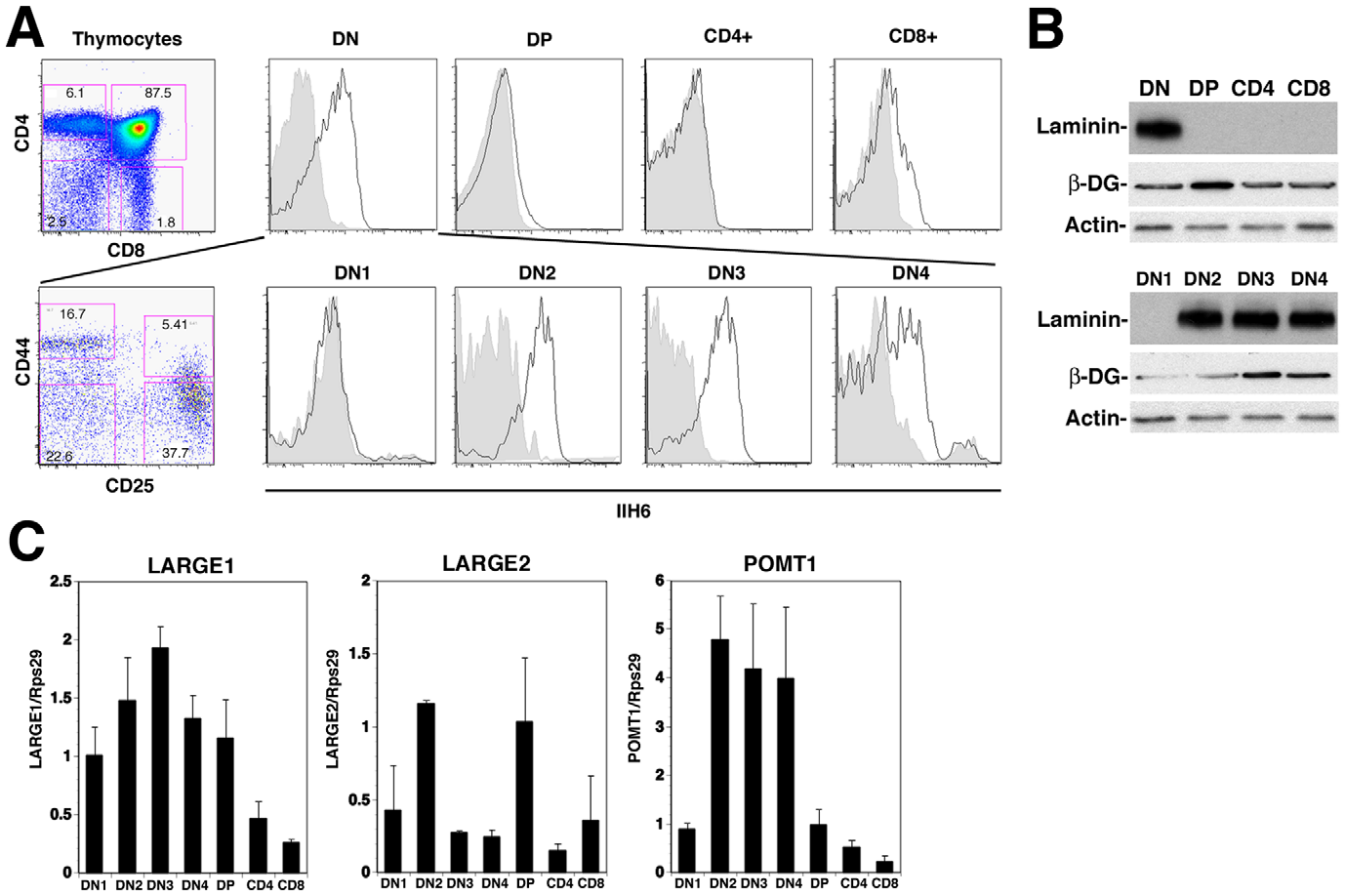
Differential Expression of Functionally Glycosylated α-DG During T Cell Development. (A) Thymi were harvested from 6- to 8-week-old C57BL/6 mice. Whole thymocyte suspensions were stained with lineage antibody cocktail (Lin, CD19/NK1.1/CD11c/TCRγδ), CD4, CD8, and IIH6 (for glycosylated α-DG). Lin^−^ cells were gated and defined the major thymocyte subsets by CD4 and CD8 as DN (CD4^−^CD8^−^), DP (CD4^+^CD8^+^), CD4^+^, and CD8^+^ T cells (left). DN lymphocytes were further segregated into various subpopulations as CD44^+^CD25^−^ DN1, CD44^+^CD25^+^ DN2, CD44^−^CD25^+^ DN3, and CD44^−^CD25^−^ DN4. Glycosylated α-DG was detected with monoclonal antibody IIH6 (right). The data represented are from at least three independent experiments. Bold line, anti-glycosylated α-DG; shaded area, unstained. (B) Thymocytes were sorted into various subsets as indicated and lysed as described in Experimental Procedures. The protein extracts were subjected to SDS-PAGE followed by laminin binding (laminin) or by immunoblotting for β-DG (core DG protein) and β-actin (loading control) expression. (C) Detection of the messages for POMT1, LARGE1, LARGE2 by quantitative RT-qPCR: Complementary DNA (cDNA) was synthesized from total RNA of each of the cell types studied. For each cell type, POMT1, LARGE1, LARGE2 and Rps29 (normalization control) were specifically amplified, in triplicate, in the presence of SYBR green. The expression of POMT1, LARGE1 and LARGE2 are shown relative to that of the Rps29 in the same sample.

To assess the biological role of α-DG in T cell development, we first examined the expression of functionally glycosylated α-DG on different subsets of thymocytes based on their expression of CD4 and CD8. Using a monoclonal antibody IIH6 specifically recognizing functionally glycosylated α-DG [18], high levels of glycosylated α-DG were detected in DN thymocytes, whereas by contrast only low levels were found on DP and both CD4^+^ and CD8^+^ single positive (SP) thymocytes (Fig. 1A, top). After sorting of DN thymocytes into the subpopulations DN1 through DN4 using the markers CD25 and CD44, we observed a significant increase of functionally glycosylated α-DG in cells progressing from DN1 to DN2 (Fig. 1A). High levels or functionally glycosylated α-DG were maintained throughout DN3 with a subsequent reduction in DN4 thymocytes (mean fluorescent intensity decreased 2–4 fold). To demonstrate the ECM binding activity of α-DG derived from different thymocyte populations, binding to laminin was assessed by overlay assay. Consistent with our flow cytometric analysis, laminin binding was detected to α-DG from DN2 through DN4 stages but not to DP and single positive thymocytes (Fig. 1B, top). Detection of DG core protein by immunoblot for β-DG revealed an increase in DG core protein expression from DN1 through DN3 and remained high in DN4, DP, CD4 and CD8 single positive cells (Fig. 1B), as well as mature splenic lymphocytes (Fig. S1). Together, the data indicate that the levels of functional α-DG during T cell development are highly regulated primarily at the level of post-translational modifications.

In order to further investigate the basis for the differential glycosylation of α-DG during T cell development, we assessed the expression levels of the known and putative glycosyltransferases POMT1/2, POMGnT1, LARGE1/2, fukutin and FKRP that are implicated in α-DG modification, using real-time quantitative PCR. Among the candidate glycosyltransferases tested, POMT1, LARGE1, and LARGE2 were subject to changes in expression during T cell development (Fig. 1C). Of particular interest is the marked induction of gene expression for POMT1 during the stages DN2 through DN4, which correlated with the increase in functional glycosylation of α-DG on T cell precursors (Fig. 1A, B). Interestingly, only mild changes in transcription of DG core protein, POMT2, POMGnT1, fukutin, and FKRP were observed in stages DN1 through DN4 (data not shown), suggesting differential regulation of the enzymes involved in α-DG glycosylation during T cell development.

### The Ablation of DG in T Cells Leads to Reduced T Cell Numbers in the Thymus and Periphery

**Figure 2:**
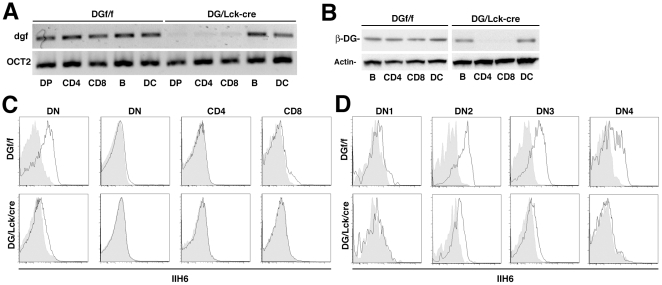
T Cell-Specific Deletion of DG in DG/Lck-cre Mice. (A) PCR analysis of *dg* locus recombination on genomic DNA from sorted DP thymocytes, splenic B cells, CD4^+^ and CD8^+^ T cells, and DCs from DG/Lck-cre mice and littermate controls (DGf/f). *Dg^f^*, floxed *dg* locus. (B) Immunoblot analysis of DG protein levels from DG/Lck-cre mice and littermate controls. (C) Flow cytometric analysis of functionally glycosylated α-DG in main subsets of thymocytes from DG/Lck-cre and control (DGf/f) mice. Bold line, anti-glycosylated α-DG; shaded area, unstained. (D) DN thymocytes were further divided into DN1∼4 on the basis of CD44 and CD25 expression and the functionally glycosylated α-DG was analyzed by flow cytometry. Representative data from one of at least four independent experiments are shown.

To investigate the roles of α-DG in T cell development and in mature T cells *in vivo*, T cell specific DG-deficient mice (DG/Lck-cre) were generated by crossing DG-floxed mice (DGf/f) with *Lck-cre* transgenic mice, an extensively used model to delete the floxed gene during T cell development [19]. The excision of floxed DG by the expression of *cre* recombinase in T cells was monitored by PCR on genomic DNA isolated from thymocytes and peripheral T cells (Fig. 2A). In line with the studies by others [19], [20], expression of Cre recombinase under the control of the Lck promoter resulted in specific recombination of the floxed DG gene in T cells, but neither B cells nor dendritic cells (Fig. 2A). The specific deletion of DG protein in splenic T cells and thymocytes were also verified by immunoblotting with an anti-β-DG antibody (Fig. 2B). As expected, ablation of the DG gene resulted in a marked reduction of α-DG in DN thymocytes (Fig. 2C). The levels of α-DG were significantly reduced in DN2 thymocytes, with a pronounced decrease in DN3, and undetectable levels in DN4 thymocytes (Fig. 2D). This result is in accordance with the progression of excision of the floxed DG allele during T cell development (Fig. S2). The marked reduction of functional α-DG in DG/Lck-cre mice on thymocytes during the crucial DN3 and DN4 stage, allowed us to use this model to address the role of DG in critical phases of early thymic T cell development, in particular at the first check point, β-selection.

**Figure 3:**
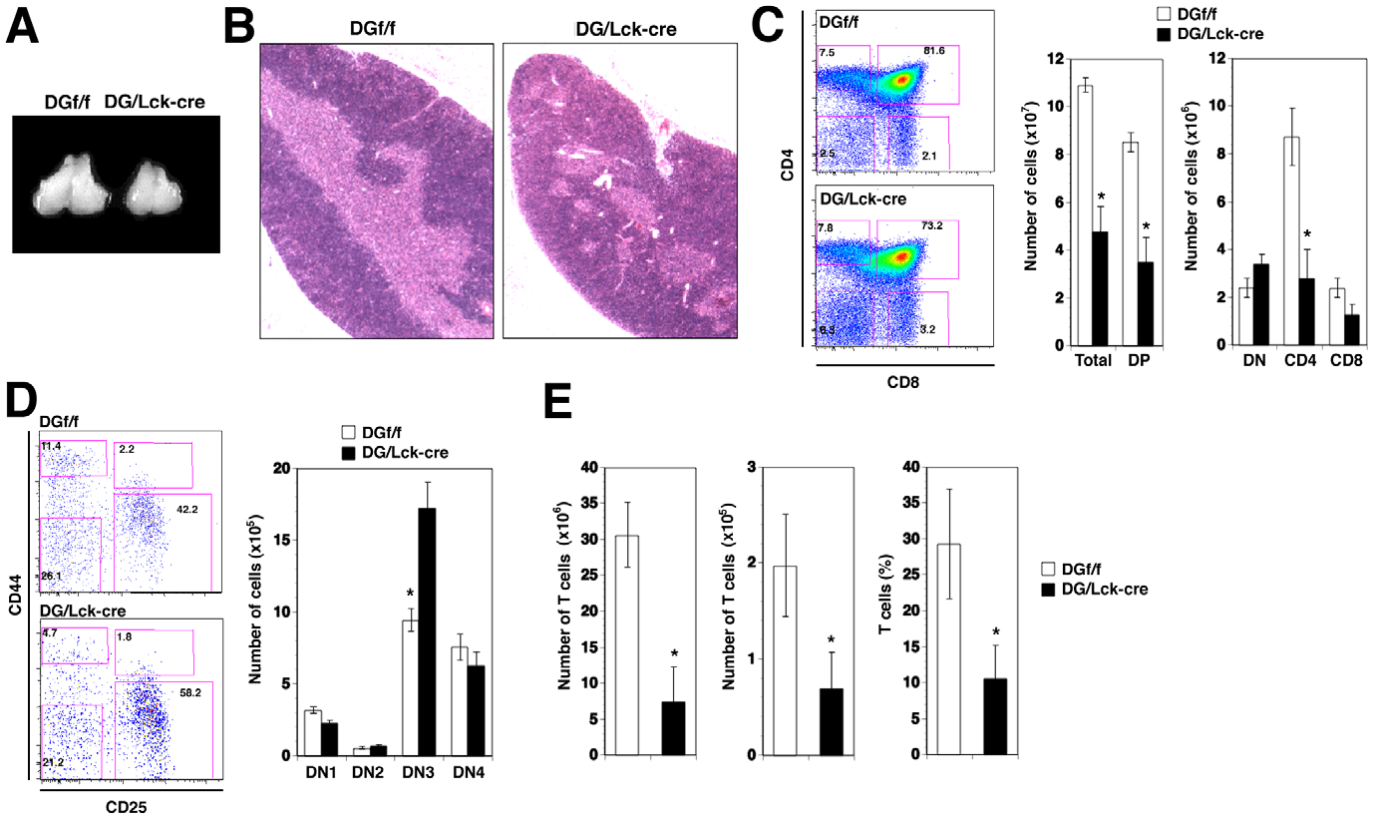
T Lymphocyte Developmental Defects in the Adult DG/Lck-cre Mice. (A) DG/Lck-cre mice had smaller thymi compared to the DGf/f controls. (B) Architecture of the thymus was assessed by hematoxylin and eosin staining on the fixed thymic sections from DG/Lck-cre and DGf/f mice. Magnification  = 5×. (C) A representative plot of CD4 vs. CD8 on gated Lin^−^ thymocytes from DG/Lck-cre and DGf/f is shown (left). Cell numbers of major thymocyte subsets from adult thymi were calculated after gating of various subsets (right). Numbers are mean ± SD from 12 mice per genotype at 6–8 wk of age. *P<0.05 (Student's two-tailed *t* test with equal variance). (D) DN subsets were assessed by CD44 and CD25 (left) and numbers of DN subsets (right) were calculated as in C. (E) Absolute cell numbers of total T cells in spleen (left) and lymph nodes (middle) from DG/Lck-cre and DGf/f are plotted. Frequency of T cells in peripheral blood is shown as compared to the control mice (right).

Next, we characterized the thymic morphology at 6–8 wks after birth in DG/Lck-cre mice compared to littermate DGf/f controls. Gross examination of thymi revealed a reduction in overall size and mass of the organ in DG/Lck-cre mice when compared to the littermate control (Fig. 3A). Histological analysis of the thymic architecture by hematoxylin and eosin (H&E) staining revealed marked hypoplasia in the thymic medulla of DG/Lck-cre mice (Fig. 3B). The absolute number of total thymocytes was reduced consistently by 50–70% in DG/Lck-cre mice (Fig. 3C, middle). Analysis of subgroups of thymocytes in DG/Lck-cre mice revealed a marked reduction in numbers of DP thymocytes with a concomitant decrease of CD4 and CD8 SP thymocytes (Fig. 3C, middle and right). In contrast, the numbers of total DN thymocytes did not significantly change. To exclude possible effects of Lck promoter driven Cre transgene on T cell development, Lck-cre mice were included as an additional control. The numbers of total thymocytes and the numbers of each thymocyte subset in Lck-cre mice were similar to the numbers observed in DGf/f mice, excluding an adverse effect of the cre transgene expression on T cell development in our system (Fig. S3). When we further dissected the different DN populations, a moderate increase in the proportion and absolute numbers of DN3 thymocytes was found in DG/Lck-cre mice (Fig. 3D). Additionally, we observed about 50–70% reduction of CD4^+^ and CD8^+^ T cells in blood, spleen, and lymph nodes in DG/Lck-cre mice (Fig. 3E), indicating that the defects in T cell development lead to the reduced T cell numbers in the periphery. However, the relative proportion of the TCR Vβ repertoire appeared comparable between the DG/Lck-cre mice and controls (Fig. S4).

### Increased Apoptosis in the Thymus of DG/Lck-cre Mice

**Figure 4:**
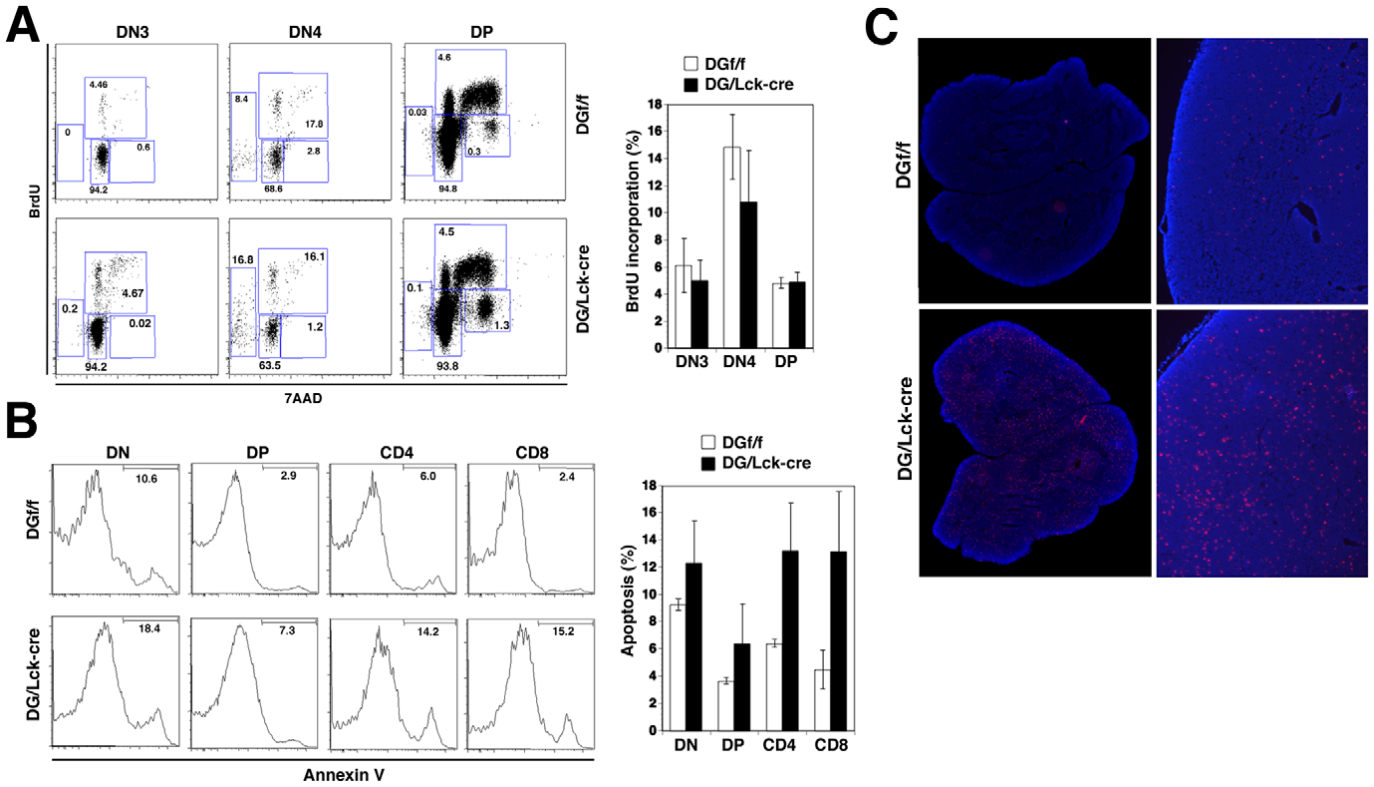
Unaffected Cell Cycle Progression but Increased Cell Death in DG/Lck-cre Mice. (A) Normal cell cycle profile in DN3, DN4, and DP thymocytes from adult thymi. Mice were inoculated with 1 mg/ml BrdU intraperitoneally 2 h before the thymus was harvested. Thymocytes were stained with the antibodies described in Materials and Methods to define thymocyte subsets. Cell cycle was analyzed by the staining with anti-BrdU antibody and 7AAD. Representative plots of BrdU vs 7AAD on gated DN3, DN4, and DP thymocytes are shown (left). Percentage of BrdU^+^ cells among DN3, DN4, and DP thymocytes are graphed (right). Data were derived from 4 mice of each genotype. (B) Apoptosis of thymocytes was analyzed by the staining with annexin V and 7AAD. Representative histograms of annexin V in various thymocyte subsets are shown (left). The percentages of annexin V^+^ cells in each subset are displayed. Representative data from one of at least four independent experiments are shown. Mean ± SD from 6 mice of each genotype. (C) *In situ* staining of apoptotic cells by TUNEL assay on the frozen thymus section. Images were taken at 5× magnification (C) and reconstructed (D) using MosaiX software.

To gain insights into the mechanisms underlying the impact of DG ablation in T cells, we first examined the proliferation of thymocytes in DG/Lck-cre mice. We injected mice with bromodeoxyuridine (BrdU) and harvested thymocytes 2 h later to analyze the incorporation of BrdU in DG/Lck-cre mice and littermate controls. The combination of anti-BrdU antibody and 7-amino-actinomycin (7-AAD) in flow cytometric analysis allows the discrimination between cell populations undergoing cell cycle progression or apoptosis. The percentage of BrdU incorporation in DN3, DN4, and DP cells were comparable between DG/Lck-cre and control mice (Fig. 4A), suggesting that proliferation (S phase) was not significantly impaired in the absence of DG. Interestingly, apoptosis (sub-G1 phase) in DN4 thymocytes of DG/Lck-cre was two to three fold higher than of controls. However, no significant apoptosis in DP thymocytes could be detected in this assay due to their high turnover rate [21]. Next we employed Annexin V staining to determine whether increased apoptosis accounts for the reduced thymocyte numbers in DG/Lck-cre mice. Indeed, significantly higher incidence of apoptosis (Annexin V positive cells) was detected in CD4 and CD8 SP thymocytes (Fig. 4B) but not in DP thymocytes likely due to their rapid turnover. These studies were complemented by *in situ* TUNEL staining to detect apoptosis in thymic tissue sections. Consistent with our findings with Annexin V staining, an overall increase in apoptotic cells was found in the thymus of DG/Lck-cre mice (Fig. 4C). Together, our data suggest that functional DG on thymocytes is not required for proliferation but is involved in survival during T cell development.

### DG/Lck-cre Mice *In Vivo* Exhibit Normal T Cell Proliferation Following Allogeneic Stimulation and Normal T Cell Functions During Either Acute LCMV or Influenza Virus Infections

**Figure 5:**
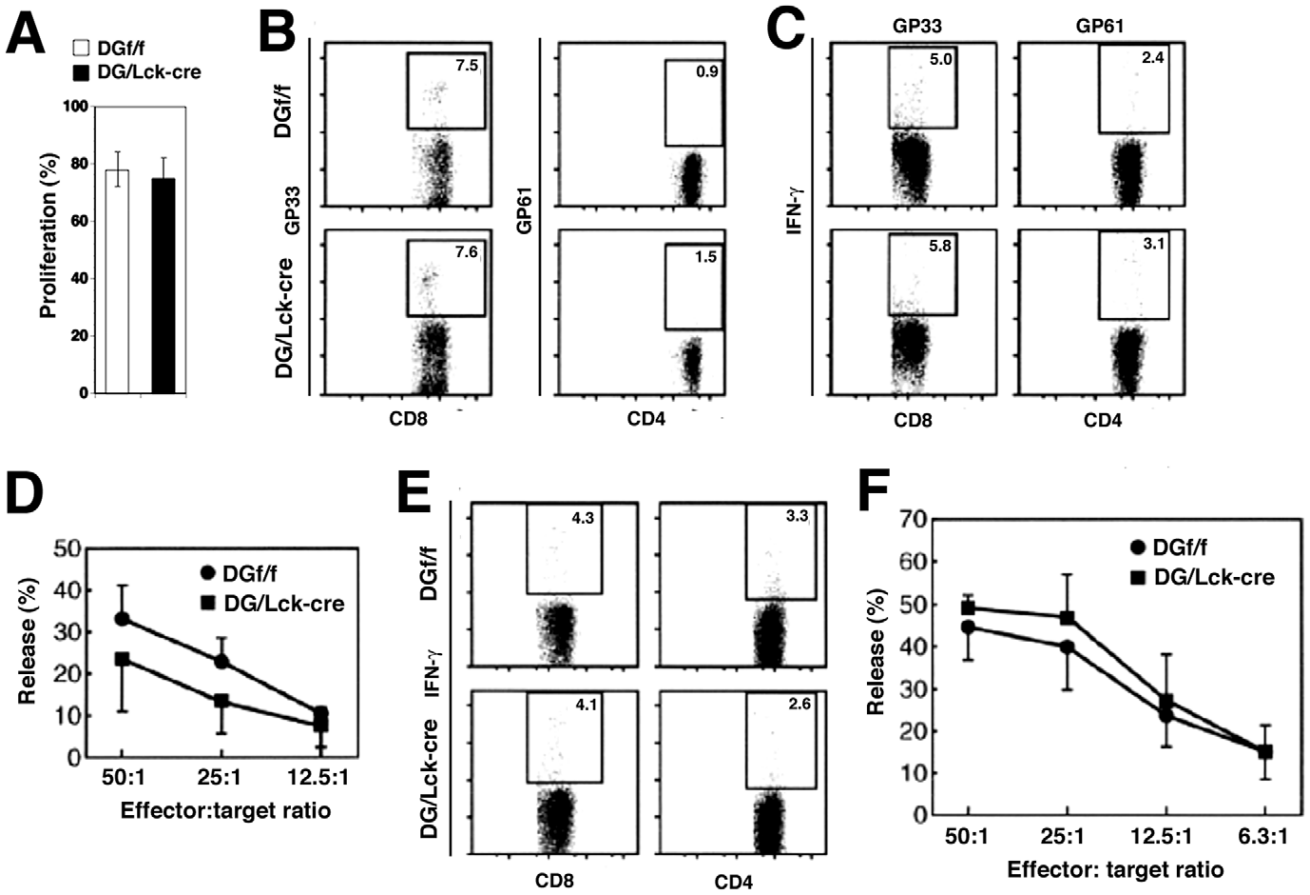
DG/Lck-cre Mice Show T Cell Responses Equivalent to Those of DGf/f Control Littermates During Allogeneic Stimulation or Acute LCMV Infection. (A) Splenic T cells were isolated from C57BL/6 (H-2b) DG/Lck-cre mice and littermate controls (DGf/f) and labeled with CFSE prior to co-culture with T cell-depleted splenocytes from Balb/c (H-2d) mice. Proliferating T cells (diluted CFSE) were assessed after staining with surface marker by flow cytometry analysis. The frequencies of proliferating T cells were quantified. (B–D) In Panels B, C and D, DG/Lck-cre mice and littermate controls (DGf/f) were inoculated intraperitoneally with LCMV ARM 1×10^5^ pfu. Eight days later, splenocytes were harvested and analyzed. (B) Panel B shows numbers of LCMV-specific T cells as determined by MHC tetramer staining. Representative data of D^b^/GP_33_ (immunodominant virus-specific CD8^+^ T cell epitope) and IA^b^/GP_61_ (immunodominant virus-specific CD4^+^ T cell epitope) are shown. (C) Panel C displays intracellular expression of IFN-γ after stimulation of splenocytes for 5 h with peptides GP33 and GP61 specific for CD8^+^ and CD4^+^ T cells respectively. Representative data from one of at least five independent experiments all with similar results. (D) Cytotoxic activity of virus-specific T cells assessed by ^51^Cr release assay. Specific lysis at varying effector to target cell ratios is shown. Values indicate the percentage of specific ^51^Cr releasing from target cells. The data represent the average and standard deviation for four mice per group. (E–F) DG/Lck-cre mice form virus specific CD8^+^ and CD4^+^ memory T cells after LCMV infection. DG/Lck-cre and DGf/f C57BL/6 mice were initially infected intraperitoneally with 1×10^5^ pfu of LCMV ARM. All mice cleared infectious virus by 15 days and at 45 days post-virus inoculation were challenged intravenously with 2×10^6^ pfu of LCMV Cl 13 to induce memory T cells [23], [30]. Spleen cells were isolated two days after Cl 13 infection. (E) In Panel E LCMV-specific T cells were determined by intracellular staining of IFN-γ producing T cells after stimulating with peptides GP33 and GP61 for 5 h, (F) while in Panel F T cell effector function was detected by ^51^Cr release assay.

Others reported a role of α-DG in mature activated T cells as a receptor for agrin at the immunological synapse *in vitro* [7], [22]. Since our DG/Lck-cre mice showed complete deletion of DG from mature naïve T cells, we studied the role of α-DG in mature activated T cells in the context of the proliferative response to allogeneic stimulation as well as the immune response to LCMV and influenza virus infections. As shown in Figure 5, panel A, splenic T cells from both DG/Lck-cre mice and control DGf/f mice displayed equivalent proliferation in response to allogeneic splenocytes, depleted of T cells, from Balb/c (H-2d) mice. To determine the T cell response of DG null mice to LCMV infection, DG/Lck-cre mice and littermate controls were challenged intraperitoneally (i.p.) with high (1×10^5^ pfu) and low (5×10^2^ pfu) doses of LCMV and then analyzed for T cell responses at day 8 post-infection, the peak phase of T cell expansion [23]. The LCMV-specific CD4^+^ and CD8^+^ T cells were detected by MHC-peptide tetramers and intracellular IFN-γ staining (Fig. 5B and 5C). DG/Lck-cre mice showed frequencies of LCMV-specific CD4^+^ and CD8^+^ T cells equivalent to that of control mice using the CD4 dominant I-A^b^-restricted epitope GP61-80 (IA^b^/GP61) and the CD8 immunodominant H-2D^b^-restricted epitope GP33-41 (Db/GP33) respectively (Fig. 5B and 5C, and Fig. S5). Likewise, determination of cytotoxic activity of CD8^+^ T cells by ^51^Cr-release assay revealed similar levels of killing by anti-viral CTLs derived from DG/Lck-cre and littermate control mice (Fig. 5D and Fig. S5). We characterized the anti-viral CD8^+^ T cell response to other dominant and subdominant H-2D^b^-restricted LCMV epitopes GP276-286, NP205-212, and NP396-404. Again, there was no apparent difference between the magnitude and quality of the anti-viral T cell response in DG/Lck-cre mice compared to control mice during acute LCMV infection (Fig. S6). Lastly, we examined whether the deletion of DG can affect the formation of anti-LCMV-specific CD8 memory T cells. To this end, we challenged LCMV ARM infected mice with a high dose of the LCMV isolate clone-13 (Cl 13) (2×10^6^ pfu) at 45 d.p.i. and assayed T cell functions 2 days later. DG/Lck-cre mice exhibited comparable LCMV specific IFN-γ expressing CD4^+^ and CD8^+^ T cells to that of littermate controls (Fig. 5E). Similar to the results we obtained in the acute LCMV infection, no significant impairment of T cell cytotoxicity was detected in DG/Lck-cre mice by ^51^Cr release assay after re-challenge (Fig. 5F and Fig. S5C), indicating a normal T memory cells response to LCMV infection in DG/Lck-cre mice.

The distribution of CD4^+^ T cell subpopulations within DG-L and DG-L/Lck-cre mice was addressed in order to detect possible differences in naïve and memory cells in mutant mice. There was no statistical difference in the distribution of naïve, as well as CD62L^+^ and CD62L^−^ I-AbGP67-77 tetramer^+^ CD4^+^ T cells within the lung, spleen and lymph nodes 45 days post-infection with LCMV-ARM (data not shown).

**Figure 6:**
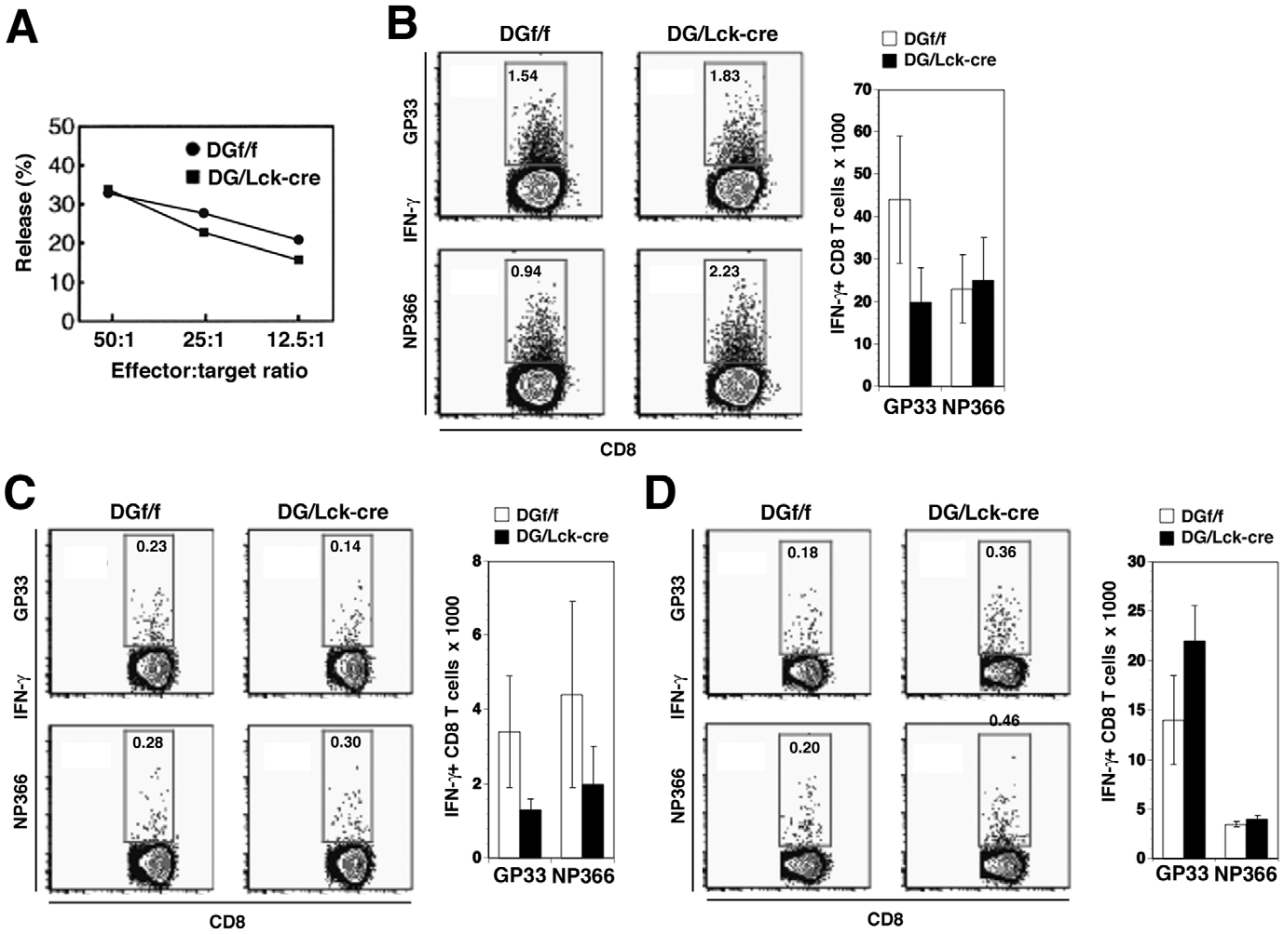
DG Null (DG/Lck-cre) Mice and Control (DGf/f) Mice Generate Equivalent Functional Virus-Specific CD8^+^ T Cell Responses After Challenge with Influenza Virus. DG/Lck-cre mice and littermate controls (DGf/f) were inoculated intratracheally with 1×10^5^ pfu of recombinant influenza virus that also expressed the immunodominant D^b^ LCMV CD8 epitope GP33. The normal influenza immunodominant epitope is NP366. (A) Eight days post-infection, the cytotoxicity of pulmonary CD8^+^ T cells was assessed by ^51^Cr release by NP366 peptide-pulsed target cells. Lungs from five mice were pooled per group. Virus-specific CD8^+^ T cells from lung (B), draining mediastinal lymph node (C) and spleen (D) were detected in mice 8 days post-infection by intracellular staining for IFN-γ following a 5 h incubation in the presence of indicated peptides. There was no statistical difference among T cell functions of DG/Lck-cre mice or DGf/f littermate controls in any of the various tissue compartments. Four to five mice used per group.

We then tested T cell responses of DG null mice to a second virus infection, i.e., response following intratracheal inoculation with recombinant influenza/LCMV virus [24]. As shown in Figure 6, Panel A, the cytotoxicity of pulmonary CD8^+^ T cells harvested from the lungs at day 8 post-intratracheal inoculation with 1×10^5^ pfu of influenza virus was equivalent in DG/Lck-cre mice when compared to DGf/f littermate controls over several effector to target ratios. Further, IFN-γ production by virus-specific CD8^+^ T cells harvested at day 8 post-intratracheal inoculation from lung, draining mediastinal lymph nodes or spleen was not significantly different between DG/Lck-cre and DGf/f mice. This was true when assaying IFN-γ responses by *ex vivo* peptide stimulation on cells from the lung (Fig. 6B), mediastinal lymph node (Fig. 6C), and spleen (Fig. 6D) to the GP33 LCMV immunodominant epitope engineered into the influenza virus neuraminidase [24] or to the H-2D^b^ immunodominant influenza NP366 epitope. Altogether, our data indicate that α-DG is dispensable for anti-T cell allogeneic proliferation and for antiviral-specific T cell effector and memory responses. Thus *in vivo* DG does not play a significant role in immunological synapse between an effector T cell and its target.

In the last series of experiments, considering the role of α-DG as an ECM receptor implicated in cell migration during the development of other tissues, e.g. the CNS [25], we addressed the possible role of α-DG in trafficking of activated T cells. To address this issue, we induced lethal lymphocytic choriomeningitis by intracerebral (i.c.) injection of LCMV into the brain of adult immunocompetent mice [26]. By this means, virus infects the leptomeninges of the brain and subsequent immunopathologic injury dependent on the migration of virus-specific cytotoxic CD8^+^ T cells into the CNS after their activation and proliferation in the periphery. Upon i.c. infection with LCMV, DG/Lck-cre mice succumbed to lethal choriomeningitis with the same kinetics as littermate controls (Fig. S7) and similar robust lymphocyte infiltration indicating that DG-deficient T cells not only expand but also migrate normally into the CNS.

## Discussion

The present study investigated the role of α-DG during T cell development and in mature T cells *in vivo*. Three main points are made. First, during the development of T cells, the expression of functionally glycosylated α-DG was differentially regulated with highest expression levels found on DN thymocytes followed by down-regulation in DP and CD4^+^ and CD8^+^ SP thymocytes. Second, ablation of DG in DN thymocytes resulted in impaired T cell development characterized by a marked reduction in cell numbers of DP and SP thymocytes associated with increased apoptosis. Third, despite the effect of α-DG on T cell development, α-DG deficient mature naïve T cells in the periphery showed normal migration, effector and memory function.

Extensive work over the past two decades has illuminated the function of DG in muscle, the nervous system, and other organs. However, the role of this important receptor in the immune system, in particular T cells, has hardly been addressed and when so studies were limited to *in vitro* systems [7]. To assess the role of DG during T cell development *in vivo*, we examined the expression of functionally glycosylated α-DG on different subsets of thymocytes using antibodies recognizing the specific sugar epitopes on α-DG that are implicated in recognition of ECM ligands and in a laminin binding assay. These studies revealed differential regulation of α-DG glycosylation during the development of T cells. Functional α-DG was detectable on early DN thymocytes with highest expression levels on thymocytes at the DN3 and DN4 stages, a critical point of transition in T cell development during which thymocytes undergo β-selection. The pattern of α-DG expression in developing T cells in the thymus of adult mice reported here varies from the results of a recent study on the dynamics of α-DG expression in T cells from fetal thymus [27]. These differences may be due to the different developmental stage of the animals used in the studies, fetal versus adult. In addition, for their FACS staining, Gong et al. [27] used a monoclonal antibody to the protein core: mAb 6C1 (mouse IgG) directed against amino acids 572–602 of human dystroglycan, whereas our studies employed mAb IIH6 (mouse IgM), which specifically recognized functionally glycosylated α-DG.

The differential expression of glycosylated α-DG during the transition between DN1 and DN4 T cell precursors correlated with a marked induction in gene expression of three known and putative gylcosyltransferases implicated in post-translational modification of α-DG, namely POMT1, LARGE1, and LARGE2. Consistent with the expression pattern of functional α-DG on T cells, ablation of the DG core protein in DN thymocytes by Cre-LoxP methodology resulted in perturbation of normal T cell development. When compared to littermate controls, mice bearing a deletion of DG in T cells had significantly reduced numbers in DP as well as CD4^+^ SP thymocytes. Using BrdU incorporation assays, we found no evidence for significant alterations in cell proliferation. However, detection of Annexin V and TUNEL staining revealed increased apoptosis of CD4 and CD8 SP thymocytes in mice lacking DG in T cells. Apoptosis in DP cells could not be addressed due to the inherently high turnover of this cell population. Our *in vivo* findings complement recent results employing re-aggregate thymus organ culture (RTOC) to demonstrate that ablation of DG in T cells *in vitro* enhanced the apoptosis rate in DP and SP thymocytes [27].

The morphological changes and marked reduction of thymocyte numbers observed in our DG/Lck-cre mice are reminiscent of the phenotype in the thymus of (dy/dy) and (dy^3k^/dy^3k^) mice that bear a genetic defect in the laminin α2 chain, a major high affinity ligand of α-DG. Like DG/Lck-cre mice, (dy/dy) and (dy^3k^/dy^3k^) mice show thymic atrophy of the outer cortex, with a marked reduction in DP cells associated with increased apoptosis [16], [28]. Interestingly, laminin isoforms containing the laminin α2 chain, laminin-2 and laminin-4, are enriched in the ECM associated with thymic epithelial cells (TEC) of the subcapsular region where DN3 cells undergo β-selection [16], [17]. The similarities between the perturbation of T cell development observed in our DG/Lck-cre mice with the defect present in mice lacking functional laminin α2 chain, together with the up-regulation of functional α-DG on DN3 and DN4 thymocytes strongly suggests that this receptor-ligand system contributes to survival of thymocytes that undergo β-selection. A role of DG in anti-apoptotic signaling is further suggested by studies in muscle cells in which blocking of laminin-α-DG binding perturbed signaling through the PI3K/Akt pathway results in increased apoptosis [29]. We are currently evaluating if a similar conserved mechanism exists for DG-mediated anti-apoptotic signaling in muscle and developing T cells.

While the perturbation of normal T cell development upon ablation of DG occurs in DN thymocytes, mature T cells in the periphery of DG/Lck-cre mice, although reduced in numbers and lacking DG, nevertheless exhibited normal T cell functions in the context of allogeneic proliferation and acute LCMV and influenza virus infections. Similar virus titers in serum were observed during LCMV infection in DG/Lck-cre mice and littermate controls and the numbers of functional anti-viral T cells by lytic assays, intracellular cytokines, migration, biologic activity in the CNS, and generation of T cell memory were comparable after intraperitoneal or intracerebral inoculations. Correspondingly, acute influenza virus infection initiated via the airways resulted in equivalent CD8^+^ CTL activity in lungs of DG/Lck-cre and control DGf/f littermates. Further, by quantitative FACS analysis and intracellular staining for IFN-γ, equivalent numbers of virus-specific CD8^+^ T cells were found in lung, mediastinal lymph nodes and spleens of influenza virus-infected DG/Lck-cre and DGf/f mice mirroring the normal migration and effector function of these cells following respiratory infection [24]. Thus, the overall normal magnitude and quality of the anti-viral T cell response in DG/Lck-cre mice and the migration pattern of such cells indicates that DG is dispensable for the normal expansion and effector function of virus-specific T cells. Further, DG was not required for formation and function of antiviral memory T cells. The lack of a phenotype despite the reduced numbers of T cells in the periphery of DG/Lck-cre mice is compatible with earlier studies that documented clearance of LCMV infection requires ∼350,000 virus specific CD8^+^ and 7,000 CD4^+^ T cells [30] and that diminished thymic input of T cells has a minimal impact on the control of LCMV infection [31].

The lack of functional impairment or migration of DG-deficient T cells in our *in vivo* studies using LCMV and influenza virus stands in contrast to earlier studies using *in vitro* co-culture systems that suggested a role of DG on T cells in the formation of functional immunological synapses between T cells and antigen-presenting cells [7], [22]. The reasons for this discrepancy are unclear but may be related to differences in the more complex *in vivo* infection model that allows for more functional redundancy and is biologically more relevant. Although our studies have not yet uncovered any overt functional defects in mature T cells lacking DG, we can at this point not categorically exclude the existence of compensatory mechanisms, e.g. changes in the selected TCR repertoire that may compensate for impaired thymocyte signaling and survival.

## Materials and Methods

### Mice

All animals were handled in strict accordance with good animal practice as defined by the requirement of the National Institute of Health and The Scripps Research Institute animal committee, and all animal work was approved by the The Scripps Research Institute animal committee. C57BL/6 or Balb/ByJ mice were bred and maintained in a closed breeding facility at the Scripps Research Institute. C57BL/6 mice bearing floxed DG alleles have been described [25]. DG/Lck-cre mice were generated by crossing the mice carrying LoxP-flanked (floxed) DG gene with *Lck-cre* transgenic mice (the Jackson Laboratory) [19].

### Genomic PCR to Detect DG Deletion

Genomic DNA from total thymocytes or sorted cells from thymus or spleen were isolated and subjected to PCR using specific primers [25] to detect the floxed and deleted DG allele. OCT2 was used as control for DNA input in PCR.

### Mononuclear Cell Isolation and Tissue Processing

The spleen, thymus, lung, and combined cervical and mediastinal lymph nodes were harvested from mice and mechanically disrupted through a 70-µm strainer. After treated with RBC lysis buffer (0.14 M NH_4_Cl in HEPES, pH 7.0), cells were washed with PBS and resuspended with complete medium (RPMI 1640 supplemented with 10% FBS, penicillin- streptomycin, HEPES, non-essential amino acid, L-glutamine, sodium pyruvate, and β-mercaptoethanol) for culture or with FACS staining buffer (2% FBS in PBS) for staining.

### Flow Cytometry and Cell Sorting

The following antibodies were purchased from either eBioscience or BD Biosciences: FITC conjugated CD3, CD4, CD44, and TNF-α antibodies, PE conjugated γδTCR, c-kit, CD19, NK1.1, and mouse-IgM antibodies, PerCP-Cy5.5 conjugated NK1.1 and CD19 antibodies, APC conjugated CD25 and IFN-γ antibodies, PE-Cy7 conjugated CD4, and Pacific Blue conjugated CD8. Cells isolated from tissue were stained for 1 h at room temperature with I-Ab*GP67*-77 tetramers (NIH tetramer core) to assess LCMV-specific CD4^+^ T cells. For intracellular staining, cells stained with surface proteins were fixed with 4% paraformaldehyde and permeabilized with saponin buffer (0.1% saponin in FACS staining buffer). Antibodies for intracellular staining were diluted and incubated with cells in the saponin buffer. Stained cells were analyzed on a LSRII flow cytometer (BD Biosciences) using DIVA software and the data were analyzed using FlowJo software (Tree Star). To sort thymocytes, non-T cell lineage cells were excluded using a mixture of PE-conjugated CD19, NK1.1, and TCRγδ antibodies. When DN subsets were desired, DP and SP thymocytes were depleted using PE-conjugated CD4 and CD8 antibodies followed by Sheep anti-Rat Dynabeads. Specific cell subsets defined by their cell surface markers were sorted by ARIA cell sorter (BD Biosciences). Sorted cells were typically >98% pure.

#### cDNA synthesis and real-time PCR assay

Total RNA was extracted from cells using the RNeasy isolation kit (Qiagen). First-strand complementary DNA (cDNA) was synthesized from total RNA using the AMV reverse transcriptase (Roche), according to the manufacturer's instructions, and a 1∶1 mix of primer random p(dN)_6_ (Roche) and oligo d(T)_ 16_ (Applied Biosystems). Each of the target genes were real-time amplified from cDNA using oligonucleotides specific to each gene (sequences and conditions available upon request), and Rps29 was used as the normalization control. The cDNA levels were determined using SYBR green in a MyiQ rt-PCR detection system (BioRad). All samples were run in triplicate.

### Cell Death and Proliferation Assay

Annexin-V and 7-AAD staining (BD Biosciences) were used to detect cell death and apoptosis followed by flow cytometric analysis. For *in vivo* proliferation, mice were injected with 1 mg of bromodeoxyuridine (BrdU, BD Biosciences) intraperitoneally. Two hours later, thymi were harvested and stained with selected antibodies followed by flow cytometric analysis. Cell cycle analysis was performed by a BrdU-FITC kit (BD Biosciences) following the manufacturer's instructions.

### TUNEL Assay

To detect apoptosis *in situ*, thymi were harvested and submerged in OCT (Tissue-Tek) and frozen on dry ice. Frozen thymi were cut in 6-µm sections and fixed with 1% paraformaldehyde. The TdT-mediated dUTP nick-end labeling (TUNEL) assay was performed using ApopTag Red kit (Millipore, Billerica, MA) following the manufacturer's protocol. All sections were costained with 1 µg/ml DAPI (Sigma-Aldrich) for 5 min at room temperature to visualize cell nuclei. The image of each field on the section was captured using an immunofluorescence microscope (Axiovert S100, Carl Zeiss MicroImaging, Inc.) with an automated xy stage, a color digital camera (Axiocam, Carl Zeiss MicroImaging, Inc.) and a 5× objective. Reconstructions were performed using the MosaiX function in KS300 image analysis software (Carl Zeiss MicroImaging, Inc.).

### Immunoblotting and Laminin Overlay Assay

Immunoblotting was performed as described [32]. In brief, sorted thymocytes were lysed in SDS-PAGE sample buffer and subjected to SDS-polyacrylamide gel electrophoresis. After transfer to a nitrocellulose membrane, membranes were blocked in 5% (wt/vol) skim milk in phosphate-buffered saline (PBS) and incubated with monoclonal antibody 8D5 to β-DG (Novocastra) and a polyclonal rabbit anti-actin antibody (ST Cruz Biotechnology). Secondary antibodies to mouse and rabbit IgG (Pierce) coupled to horse radish peroxidase (HRP) were applied 1∶5,000 in PBS-0.1% (wt/vol) Tween 20 for 1 h at room temperature. Blots were developed by enhanced chemiluminescence (ECL) using Super Signal West Pico ECL substrate (Pierce). Laminin overlay assay (LOA) was performed as described [33].

### Virus and Infection

LCMV Armstrong clone 53b (ARM) and Clone 13 (Cl 13) were plaque-purified three times on Vero cells and stocks prepared by a single passage on BHK-21 cells [34]. Viral titers were determined by plaque formation on Vero cells [34]. Mice at the age of 6–8 weeks were injected intraperitoneal (i.p.) with 1×10^5^ or 5×10^2^ plaque-forming units (pfu). For the rechallenge experiments, mice were infected intravenously (i.v.) with 2×10^6^ pfu of LCMV Cl 13. For lethal choriomeningitis, mice were inoculated with 10^3^ pfu of LCMV ARM intracranially (i.c.). For determination of LCMV-specific CD4^+^ T cell memory, mice were infected with 2×10^6^ PFU LCMV-ARM i.v. Influenza virus (A/WSN/33; H1N1) recombinant expressing the LCMV H-2^b^ immunodominant CD8 and CD4 T cell epitopes engineered into the influenza neuraminidase stalk was used as described [24]. Briefly, DG/Lck-cre mice and littermate controls (DGf/f) were inoculated intratracheally with the influenza recombinant virus using 1×10^5^ pfu. After 8 days of infection, the cytotoxicity of T cells was assessed by ^51^Cr-release assay while viral-specific CD8 and CD4 T cells were detected by incubation with various peptides as indicated below.

### Cytotoxicity (CTL) Assay

Virus-specific CTL lysis was quantitated with a ^51^Cr-release assay as described [35]. In brief, splenocytes from LCMV ARM or splenocytes, lymph node and pulmonary cells from recombinant influenza virus infected mice at 8 d.p.i. were obtained. For LCMV, BALB Cl 7 (H-2^d^) and MC57 (H-2^b^) target cells infected with LCMV were labeled with ^51^Cr. E/T ratios of 50∶1, 25∶1, 12.5∶1, and 6.3∶1 were used. For influenza virus, ^51^Cr labeled MC57 cells target cells were loaded with influenza virus peptide NP366 (ASNENMDAM) for 1 hr at 37°C. After 5 h incubation, cell supernatant was collected and quantitated by a 20/20 series γ-counter. All samples were run in triplicate, and results were calculated as: 100× (experimental release-spontaneous release)/(maximum release-spontaneous release).

### T Cell Assay

For intracellular cytokine analysis of LCMV-infected mice, T cells were isolated after 8 days post infection and stimulated for 5 hours with 5 µg/ml of the MHC class II-restricted LCMV-GP61-80 peptide or 2 µg/ml of individual MHC class I-restricted LCMV-GP33–41, LCMV-GP276–286, LCMV-NP396–404, LCMV-NP205–212 peptides (all >99% pure; Synpep Corp.) in the presence of 50 U/ml recombinant IL-2 (R&D systems) and 1 µg/ml brefeldin A (Sigma-Aldrich). For tetramer staining, D^b^GP33 (MHC class I) and I-A^b^GP61 (MHC class II) tetramers were used at a 1∶50–1∶100 dilution in FACS buffer. For analysis of CD8^+^ T cell intracellular cytokine expression, cells form lung, mediastinal lymph node, and spleen were incubated with 2 µg/ml of individual MHC class I-restricted LCMV-GP33–41 or influenza virus-NP366 in the presence of 50 U/ml recombinant IL-2 (R&D systems) and 1 µg/ml brefeldin A (Sigma-Aldrich) for 5 hr at 37°C.

### Mixed Leukocyte Reaction

Naïve splenocytes were harvested from DGf/f and DG/Lck-cre mice (H-2^b^). T cells were purified using the EasySep® T Cell Enrichment Kit (Stemcell Technologies Inc.). Purified T cells were labeled with CFSE (Invitrogen) to monitor proliferation. Antigen-presenting cells (APC) were purified from Balb/c splenocytes (H-2^d^) using the EasySep® Mouse CD90 Positive Selection Kit (Stemcell Technologies Inc.). DGf/f and DG/Lck-cre T cells labeled with CFSE were co-cultured with Balb/c APC in a 96-well U-bottom plate at a ratio of 4∶1 (2×10^5^ Balb/c APC: 5×10^4^ T cells). Cultures were placed at 37°C at 5% CO_2_ for 5 days. Cell proliferation was analyzed by flow cytometry assessing dilution of CFSE within total T cells.

## Supporting Information

Figure S1 Resting T cell express core but not functionally glycosylated α-DG. Splenocytes were isolated from C57BL/6 mice and analyzed by flow cytometry. Glycosylated α-DG and the core α-DG were detected with monoclonal antibodies and shown on gated CD3+ T cells. Bold line, anti-glycosylated or core α-DG as indicated; shaded area, isotype control.
(0.55 MB TIF)

Figure S2 Ablation of DG gene in DG/Lck-cre mice during T cell development. PCR were performed on genomic DNA for sorted thymocyte subsets from DG/Lck-cre mice for recombined DG locus (dgΔ) and OCT2 (control).
(0.34 MB TIF)

Figure S3 Both Lck-cre and control mice show comparable numbers of thymocytes. Thymocytes were harvested from DGf/f (littermate control), Lck-cre, and DG/Lck-cre mice, then stained with specific antibodies as described in the Materials and Methods followed by flow cytometric analysis. Cell numbers were calculated from the total thymocytes versus the frequency of each thymocyte subset.
(0.20 MB TIF)

Figure S4 DG/Lck-cre mice and littermate control (DGf/f) mice express comparable TCR Vβ repertoire in splenic T cells. Splenocytes from DG/Lck-cre and DGf/f mice were stained with antibodies to surface markers and TCR Vβ chain and subjected to flow cytometric analysis. The percentage of TCR Vβ-positive cells was analyzed by gating on CD4+ and CD8+ T cells.
(0.43 MB TIF)

Figure S5 Both DG/Lck-cre mice and DG sufficient (DGf/f) mice generate functional virus specific T cell responses after challenged with low dose of LCMV ARM. DG/Lck-cre mice and littermate controls (DGf/f) were inoculated with LCMV-ARM 5×102 pfu intraperitoneally. A. After 8 days of infection, viral specific CD8+ and CD4+ T cells were detected by incubation with peptides GP33 and GP61 respectively. IFN-γ expressing cells were detected by intracellular staining. B. Detection of memory T cell response after 2 days of challenging with LCMV-Cl 13. C. The cytotoxicity of T cells was assessed by 51Cr releasing assay. See materials and methods for details.
(3.30 MB TIF)

Figure S6 LCMV infection generates virus specific CD8 T cell responses to both dominant and sub-dominant epitopes in DG/Lck-cre and control (DGf/f) mice. Splenocytes were isolated and stimulated with various dominant and sub-dominant LCMV-specific peptides for 5 hours as described in materials and methods. Intracellular staining to IFN-γ was performed to reveal LCMV specific CD8+ T cells. Representative data are shown from one of three independent experiments with at least four mice for each genotype.
(6.71 MB TIF)

Figure S7 DG/Lck-cre mice and control DGf/f littermates show comparable kinetics in developing LCMV induced meningitis. DG/Lck-cre mice and littermate controls (DGf/f) were inoculated with LCMV ARM 1×103 pfu intracranially and the survival rates of infected mice were plotted. Representative data are derived from 6 mice per genotype.
(1.40 MB TIF)
