# Supplementary figures and images for: Functional Glycosylation of Dystroglycan Is Crucial for Thymocyte Development in the Mouse
Source: PLoS One. 2010 Mar 29;5(3):e9915. doi: 10.1371/journal.pone.0009915 (PMC2848029; doi:10.1371/journal.pone.0009915)

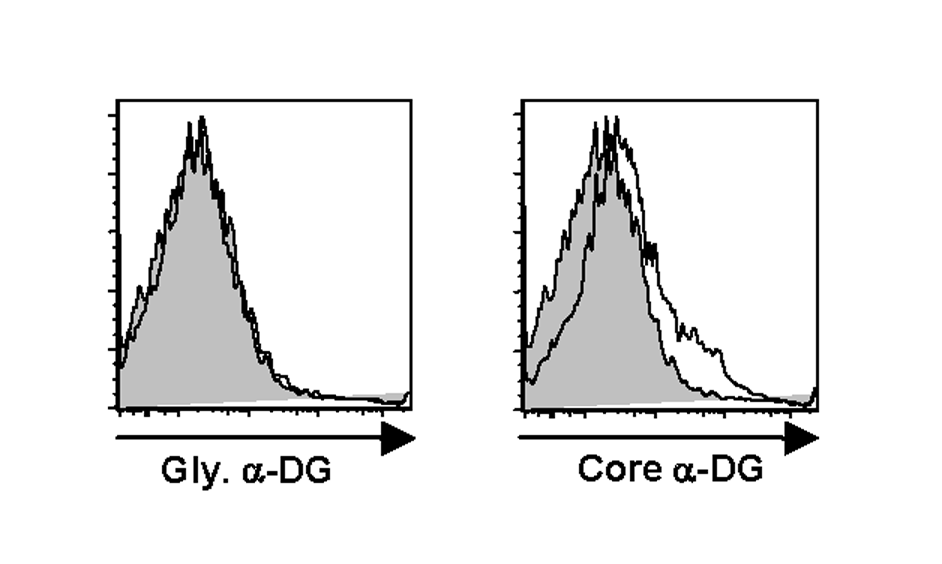

Supplement: Figure S1 — Resting T cell express core but not functionally glycosylated α-DG. Splenocytes were isolated from C57BL/6 mice and analyzed by flow cytometry. Glycosylated α-DG and the core α-DG were detected with monoclonal antibodies and shown on gated CD3+ T cells. Bold line, anti-glycosylated or core α-DG as indicated; shaded area, isotype control. (0.55 MB TIF) [file pone.0009915.s001.tif]

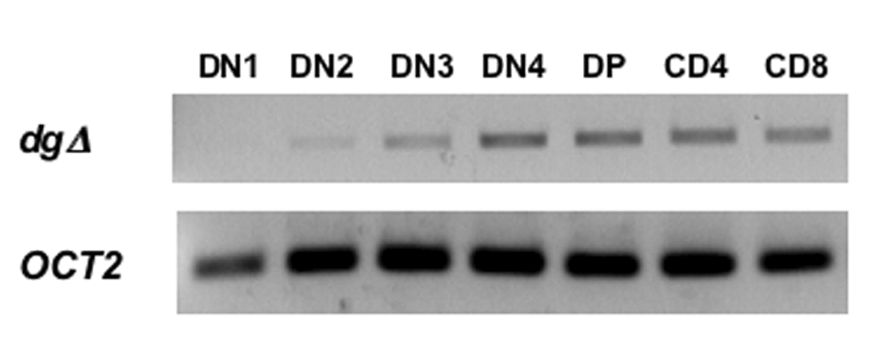

Supplement: Figure S2 — Ablation of DG gene in DG/Lck-cre mice during T cell development. PCR were performed on genomic DNA for sorted thymocyte subsets from DG/Lck-cre mice for recombined DG locus (dgΔ) and OCT2 (control). (0.34 MB TIF) [file pone.0009915.s002.tif]

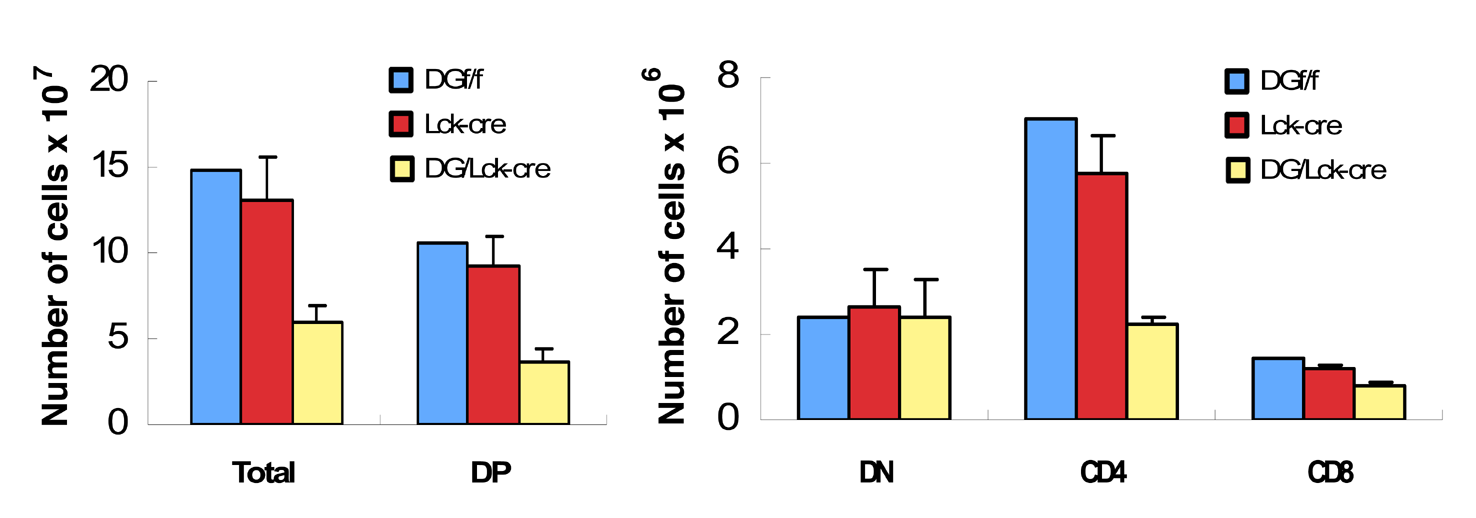

Supplement: Figure S3 — Both Lck-cre and control mice show comparable numbers of thymocytes. Thymocytes were harvested from DGf/f (littermate control), Lck-cre, and DG/Lck-cre mice, then stained with specific antibodies as described in the Materials and Methods followed by flow cytometric analysis. Cell numbers were calculated from the total thymocytes versus the frequency of each thymocyte subset. (0.20 MB TIF) [file pone.0009915.s003.tif]

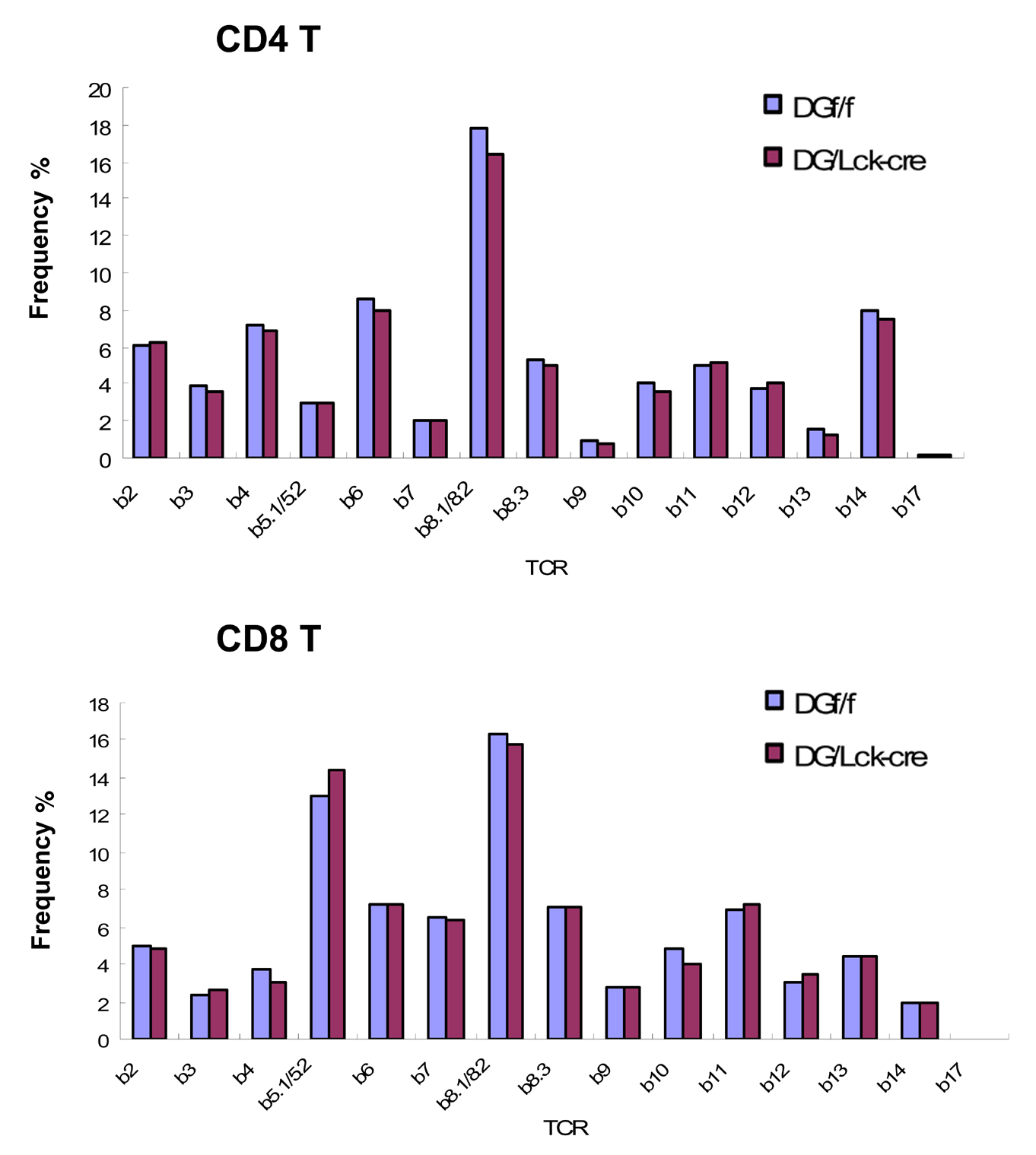

Supplement: Figure S4 — DG/Lck-cre mice and littermate control (DGf/f) mice express comparable TCR Vβ repertoire in splenic T cells. Splenocytes from DG/Lck-cre and DGf/f mice were stained with antibodies to surface markers and TCR Vβ chain and subjected to flow cytometric analysis. The percentage of TCR Vβ-positive cells was analyzed by gating on CD4+ and CD8+ T cells. (0.43 MB TIF) [file pone.0009915.s004.tif]

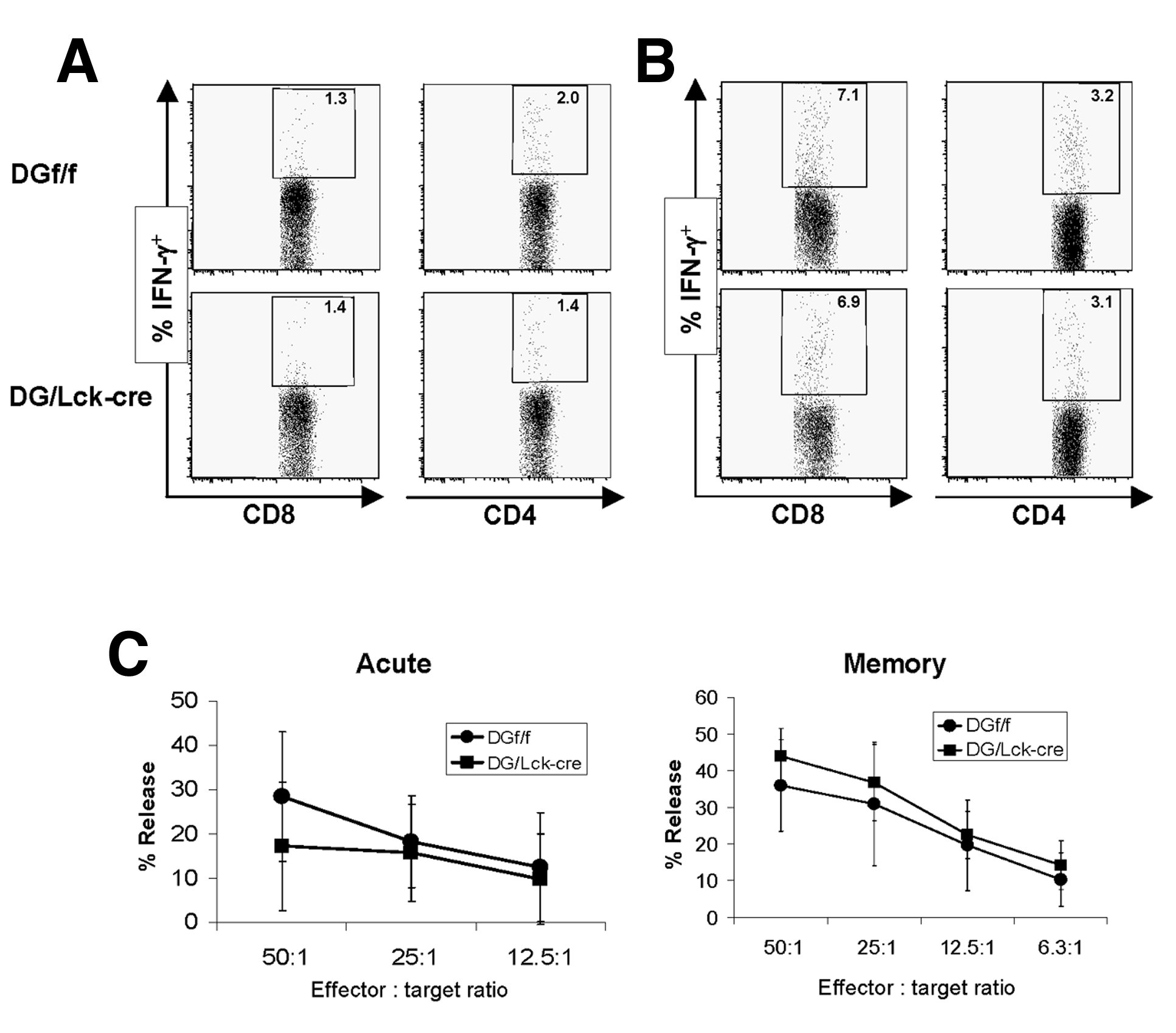

Supplement: Figure S5 — Both DG/Lck-cre mice and DG sufficient (DGf/f) mice generate functional virus specific T cell responses after challenged with low dose of LCMV ARM. DG/Lck-cre mice and littermate controls (DGf/f) were inoculated with LCMV-ARM 5×10^2^ pfu intraperitoneally. A. After 8 days of infection, viral specific CD8+ and CD4+ T cells were detected by incubation with peptides GP33 and GP61 respectively. IFN-γ expressing cells were detected by intracellular staining. B. Detection of memory T cell response after 2 days of challenging with LCMV-Cl 13. C. The cytotoxicity of T cells was assessed by 51Cr releasing assay. See materials and methods for details. (3.30 MB TIF) [file pone.0009915.s005.tif]

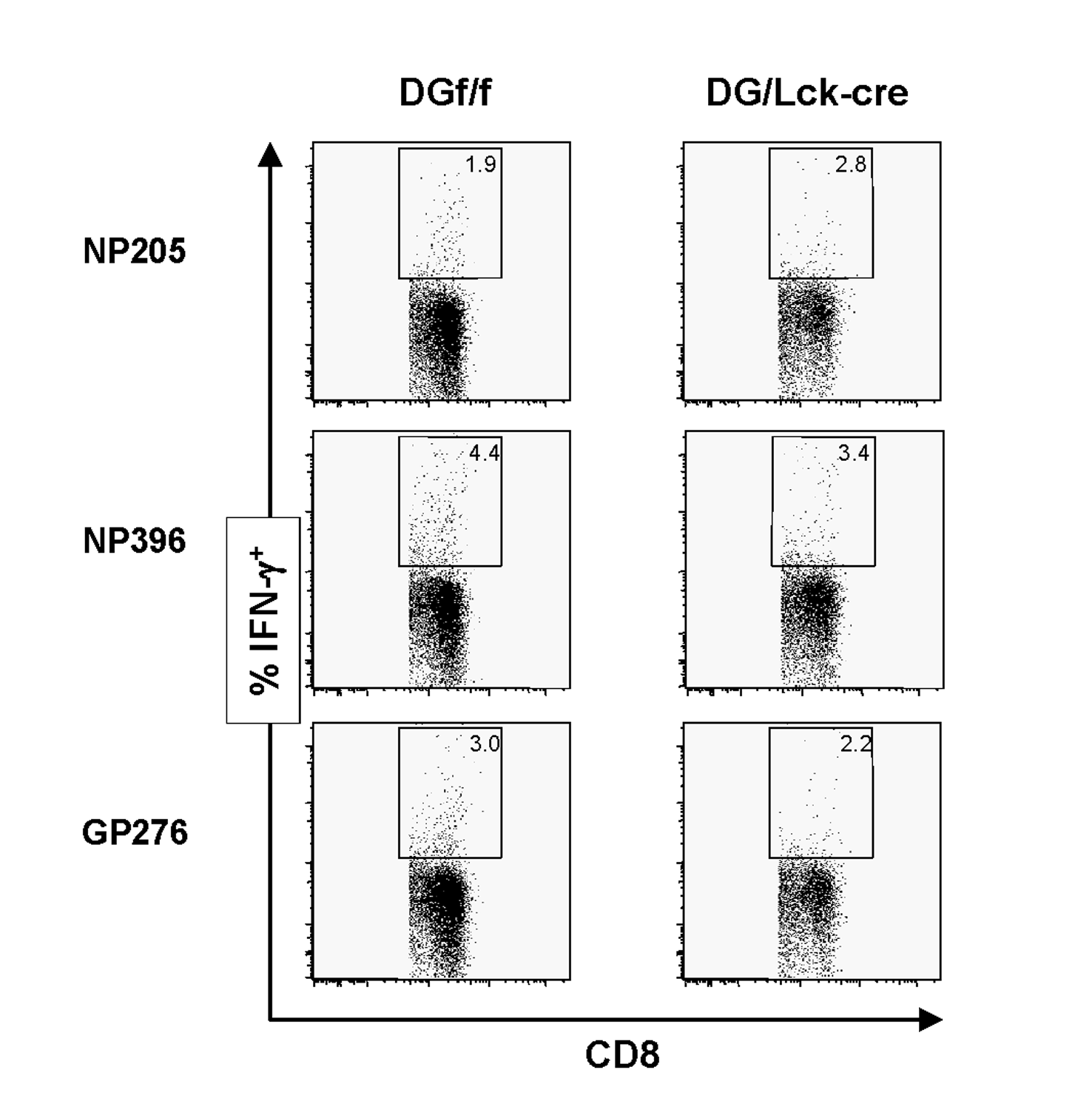

Supplement: Figure S6 — LCMV infection generates virus specific CD8 T cell responses to both dominant and sub-dominant epitopes in DG/Lck-cre and control (DGf/f) mice. Splenocytes were isolated and stimulated with various dominant and sub-dominant LCMV-specific peptides for 5 hours as described in materials and methods. Intracellular staining to IFN-γ was performed to reveal LCMV specific CD8+ T cells. Representative data are shown from one of three independent experiments with at least four mice for each genotype. (6.71 MB TIF) [file pone.0009915.s006.tif]

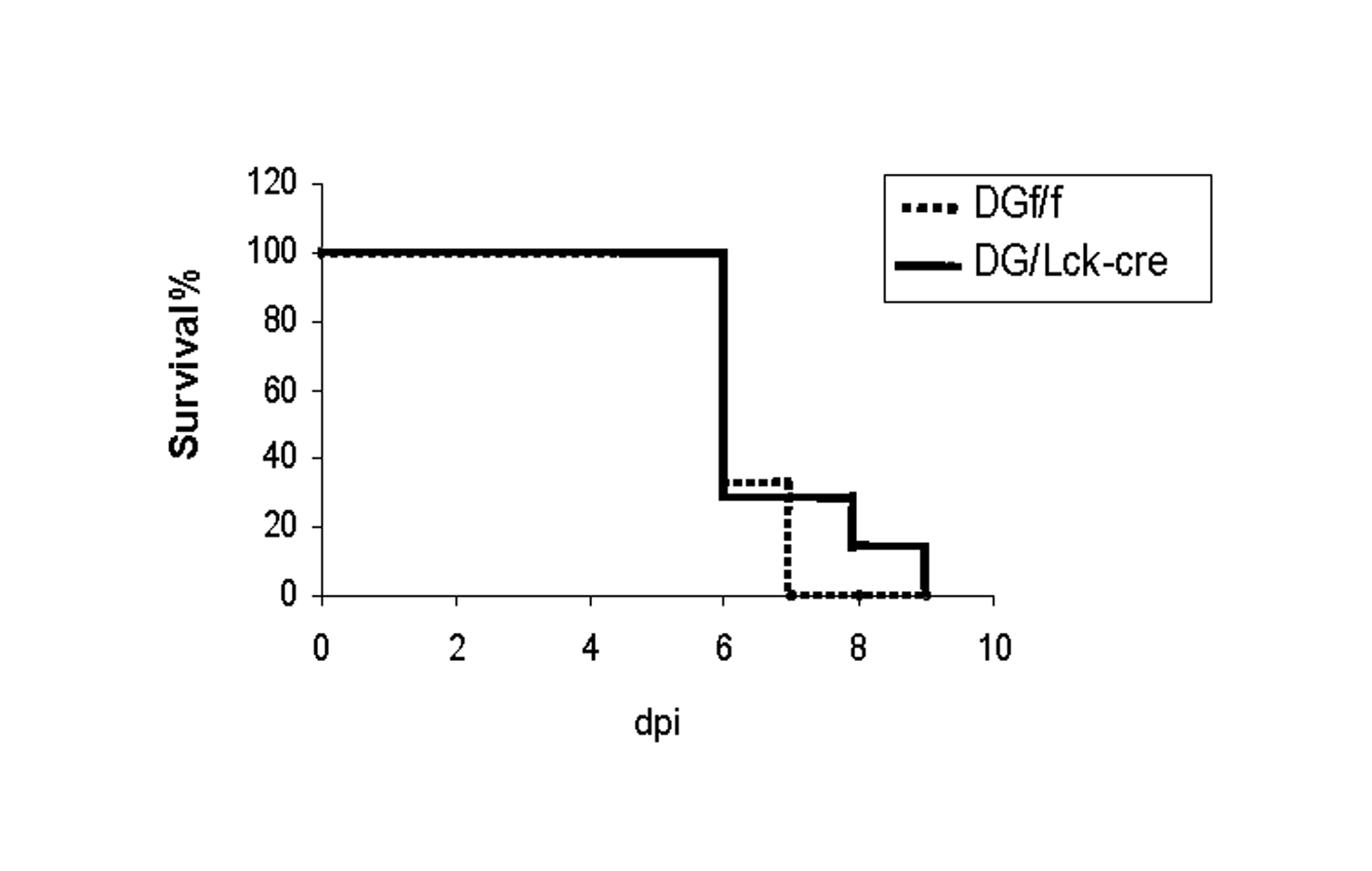

Supplement: Figure S7 — DG/Lck-cre mice and control DGf/f littermates show comparable kinetics in developing LCMV induced meningitis. DG/Lck-cre mice and littermate controls (DGf/f) were inoculated with LCMV ARM 1×10^3^ pfu intracranially and the survival rates of infected mice were plotted. Representative data are derived from 6 mice per genotype. (1.40 MB TIF) [file pone.0009915.s007.tif]
